# Supplementary material for: ‘Garlic-lipo’4Plants: Liposome-Encapsulated Garlic Extract Stimulates ABA Pathway and PR Genes in Wheat (Triticum aestivum)
Source: Plants (Basel). 2023 Feb 7;12(4):743. doi: 10.3390/plants12040743 (PMC9962754; doi:10.3390/plants12040743)
Supplement: Supplementary file 1 [file plants-12-00743-s001.zip › plants-2180331-supplementary.pdf]

Supplementary (Kutasy et al. 2023)

Figure S1. Heatmap of Top50 differentiated gene expression (DEG) based on the comparison of 15min vs. control samples. The heatmap colour range is from red for positive Z-scores values to green for Z-score negative values. Number of differentially expressed (DE) features (Probability > 0.9): 1,712. Up-regulated (M > 0): 919. Down-regulated (M < 0): 793. Annotation of transcript IDs see in AnnotationTable (DOI: 10.17632/p66v4yxbtp.1).

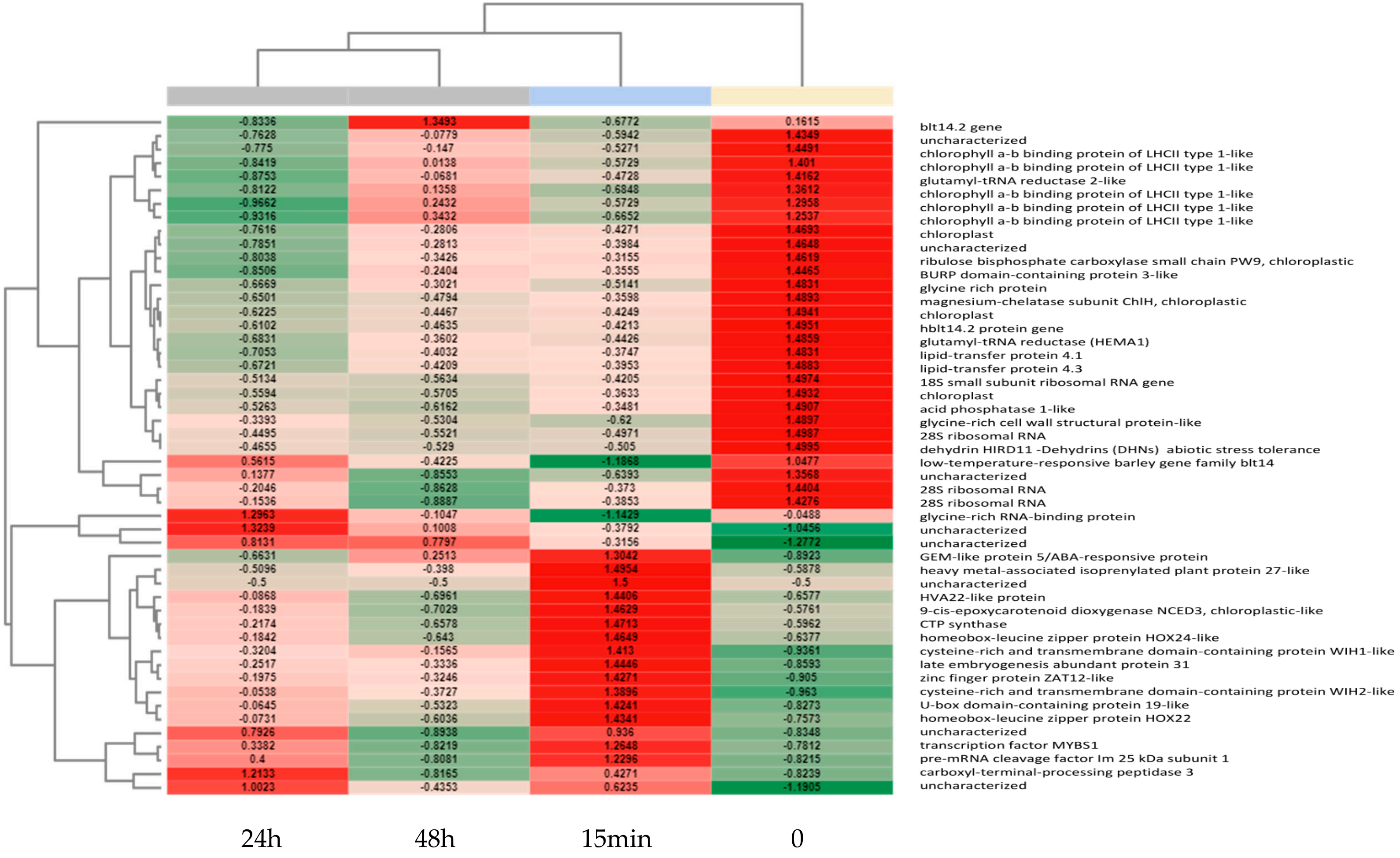

Figure S2. Heatmap of Top50 differentiated gene expression (DEG) based on the comparison of 24h vs. control samples. The heatmap colour range is from red for positive Z-scores values to green for Z-score negative values. Number of differentially expressed (DE) features (Probability > 0.9): 2,485. Up-regulated (M > 0): 1,279. Down-regulated (M < 0): 1,206. Annotation of transcript IDs see in AnnotationTable (DOI: 10.17632/p66v4yxbtp.1).

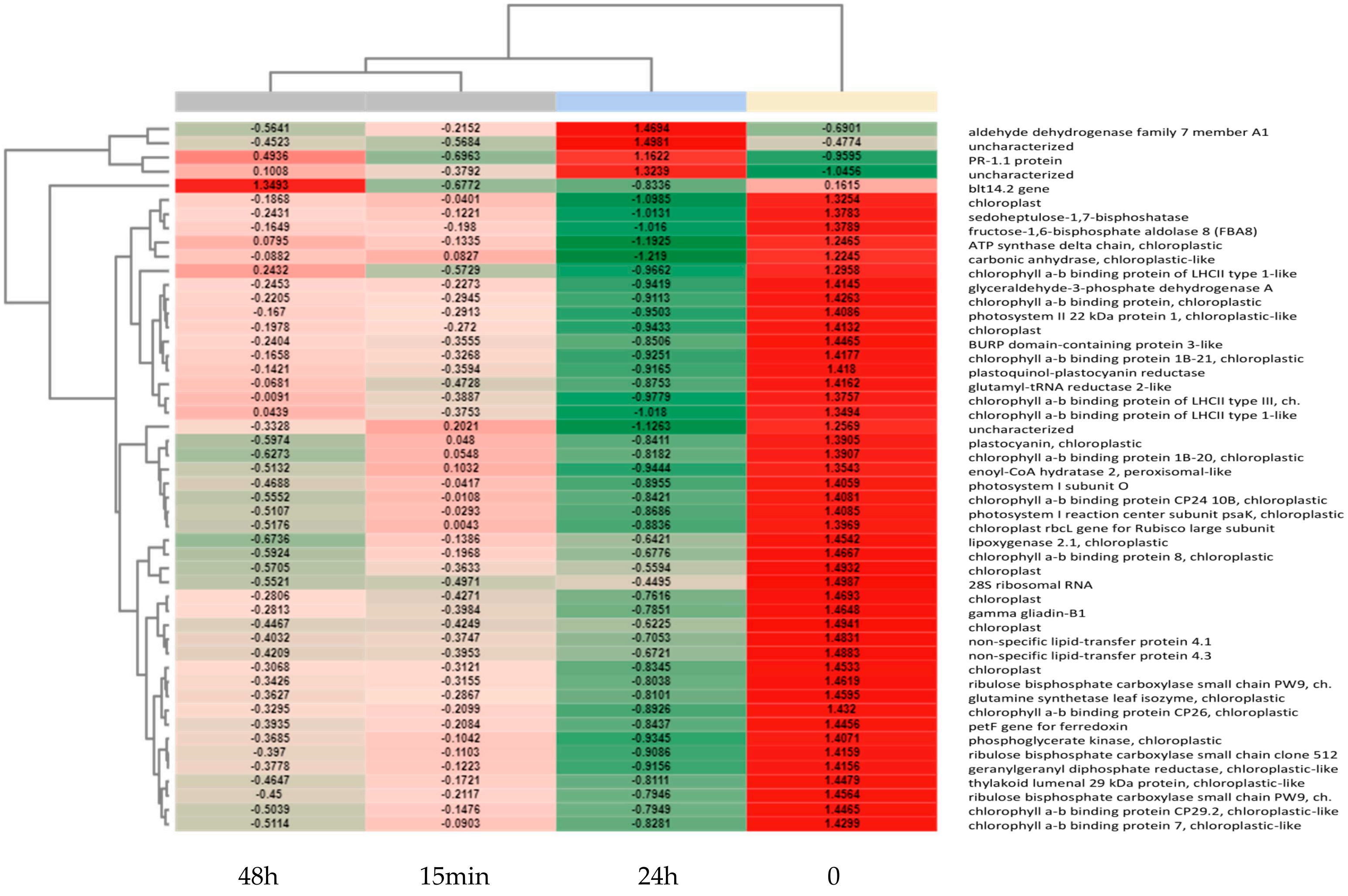

Table S1. Time-course expression analysis (DOI: 10.17632/xvvscxpz6w.1) of count data (using CoutTable DOI: 10.17632/xvvscxpz6w.1) was performed to detect genomic features with significant temporal expression changes and were divided into nine clusters. Differentially expressed features identified (False Discovery Rate < 0.05): 2,856. Significant features (R-squared > 0.7) according to the selection procedure: All significant features: 1,046. Significant features for each regression variable: START: 1,042; LINEAR: 689 and CURVED: 663. The genes of the nine clusters were classified also by Kyoto Encyclopedia for Genes and Genomes (KEGG) analysis and were supported by literature publications (DOI marked per genes).

cluster 1	Pathway	Enzymes Codes	Enzymes Name	KEGG Orthologs	KEGG Orthologs Name
1	Diterpenoid biosynthesis	ec:1.14.14.86	ent-kaurene monooxygenase 10.1104/pp.119.2.507	ko:K21719; ko:K04122	ent-sandaracopimaradiene 3-hydroxylase (CYP701A8) 10.1007/s00253-015-6496-2; ent-kaurene oxidase (CYP701) 10.1073/pnas.95.15.9019
2	Plant hormone signal transduction			ko:K13449	pathogenesis-related protein 1 (PR1) 10.1016/j.micres.2018.04.008
3	Monoterpenoid biosynthesis			ko:K21374	7-deoxyloganetin glucosyltransferase (UGT85A23_24) 10.1074/jbc.M111.242586
4	Stilbenoid, diarylheptanoid and gingerol biosynthesis			ko:K13065; ko:K16040	shikimate O-hydroxycinnamoyltransferase (HCT) 10.1105/tpc.020297; trans-resveratrol di-O-methyltransferase (ROMT)10.1104/pp.108.126003
5	Glycosphingolipid biosynthesis	ec:2.4.1.69	galactoside alpha-(1,2)-fucosyltransferase		
6	MAPK signaling pathway - plant			ko:K13449	pathogenesis-related protein 1 (PR1) 10.1016/j.micres.2018.04.008
7	Polyketide sugar unit biosynthesis	ec:4.2.1.46	dTDP-glucose 4,6-dehydratase 10.1139/b02-088		
8	Flavonoid biosynthesis			ko:K13065	shikimate O-hydroxycinnamoyltransferase (HCT) 10.1105/tpc.020297
9	Phenylpropanoid biosynthesis	ec:2.1.1.68	caffeate O-methyltransferase 10.1016/0304-4165(74)90137-8 10.1111/j.1438-8677.1999.tb00256.x	ko:K13065; ko:K00430;	shikimate O-hydroxycinnamoyltransferase (HCT); peroxidase;

				ko:K13066	caffeic acid 3-O-methyltransferase (COMT) 10.1111/jpi.12160
10	Pyruvate metabolism	ec:1.1.1.39; ec:1.1.1.38	malate dehydrogenase 10.1104/pp.90.2.367 10.1111/pbi.12556		
11	Ether lipid metabolism	ec:3.1.1.47	1-alkyl-2-acetylglycerophosphocholine esterase		
12	Amino sugar and nucleotide sugar metabolism	ec:3.2.1.14	chitinase 10.1080/07352689.2011.616043		
13	Porphyrin and chlorophyll metabolism	ec:3.1.1.14	chitinase 10.1080/07352689.2011.616043		
14	Metabolism of xenobiotics by cytochrome P450	ec:2.5.1.18	glutathione S-transferase 10.3389/fpls.2018.01836	ko:K00799	glutathione S-transferase (GST) 10.3389/fpls.2018.01836
15	Glycerolipid metabolism	ec:1.1.1.21	aldose reductase 10.1007/s11627-000-0022-6		
16	Glutathione metabolism	ec:2.5.1.18; ec:1.11.1.11	glutathione transferase 10.3389/fpls.2018.01836; L-ascorbate peroxidase10.1271/bbb.80062	ko:K00799	glutathione S-transferase (GST) 10.3389/fpls.2018.01836
17	Pentose and glucuronate interconversions	ec:1.1.1.21	aldose reductase 10.1007/s11738-013-1445-0		
18	Tryptophan metabolism			ko:K13066	caffeic acid 3-O-methyltransferase 10.1111/jpi.12160
19	Nitrogen metabolism	ec:1.13.12.16	nitronate monooxygenase 10.1371/journal.pone.0195488		
20	Carbon fixation in photosynthetic organisms	ec:1.1.1.39	malate dehydrogenase 10.1104/pp.90.2.367 10.1093/jxb/err386		

21	Plant-pathogen interaction			ko:K13449; ko:K13457	pathogenesis-related protein 1 (PR1) 10.1016/j.micres.2018.04.008; resistance protein (RPM1) 10.1105/tpc.010393
22	Ascorbate and aldarate metabolism	ec:1.1.1.122; ec:1.11.1.11	D-threo-aldose 1-dehydrogenase 10.1007/s13562-021-00714-9; L-ascorbate peroxidase 10.3389/fpls.2017.02262		
23	Galactose metabolism	ec:1.1.1.21	aldose reductase 10.1007/s11627-000-0022-6		
24	Fructose and mannose metabolism	ec:1.1.1.122; ec:1.1.1.21	D-threo-aldose 1-dehydrogenase 10.1007/s13562-021-00714-9; aldose reductase 10.1007/s11627-000-0022-6		
cluster 2	Pathway	Enzymes Codes	Enzymes Name	KEGG Orthologs	KEGG Orthologs Name
1	Ribosome			ko:K02924	large subunit ribosomal protein L39e (RPL39) 10.1111/tpj.15667
2	Glycerophospholipid metabolism	ec:2.3.1.15	glycerol-3-phosphate 1-O-acyltransferase 10.1104/pp.68.3.653 10.1104/pp.112.201996		
3	Porphyrin and chlorophyll metabolism	ec:1.14.13.122	chlorophyllide-a oxygenase 10.1046/j.1365-313x.2000.00672.x		
4	Aminoacyl-tRNA biosynthesis			ko:K01886	glutaminyl-tRNA synthetase 10.1016/S0167-4781(98)00113-4 10.3389/fpls.2015.00735
5	Metabolism of xenobiotics by cytochrome P450	ec:2.5.1.18	glutathione S-transferase 10.3389/fpls.2018.01836		
6	Glycerolipid metabolism	ec:2.3.1.15	glycerol-3-phosphate 1-O-acyltransferase 10.1104/pp.112.201996		
7	Purine metabolism	ec:2.7.4.6	nucleoside-diphosphate kinase 10.1007/s00425-014-2161-8		
8	Pyrimidine metabolism	ec:2.7.4.6	nucleoside-diphosphate kinase		

9	Tyrosine metabolism	ec:5.2.1.2	maleylacetoacetate isomerase 10.1016/j.plantsci.2006.04.008		
10	Glutathione metabolism	ec:2.5.1.18	glutathione S-transferase 10.3389/fpls.2018.01836		
11	Cysteine and methionine metabolism	ec:2.1.1.14	S-methyltransferase 10.1111/j.1432-1033.1995.1053g.x		
cluster 3	Pathway	Enzymes Codes	Enzymes Name	KEGG Orthologs	KEGG Orthologs Name
1	Biosynthesis of cofactors			ko:K00059	3-oxoacyl-[acyl-carrier protein] reductase (OAR1) 10.1016/j.scienta.2020.109388
2	Phenylpropanoid biosynthesis	ec:1.1.1.195	cinnamyl-alcohol dehydrogenase 10.1093/jxb/erq107 10.3389/fpls.2016.01723		
3	Pyruvate metabolism	ec:1.1.1.79	glyoxylate reductase (NADP+) 10.1093/jxb/ern122		
4	Glycerophospholipid metabolism	ec:2.1.1.103	phosphoethanolamine N-methyltransferase 10.1074/jbc.M109.022657	ko:K05929	phosphoethanolamine N-methyltransferase (NMT) 10.1074/jbc.M109.022657
5	Amino sugar and nucleotide sugar metabolism	ec:2.4.1.43; ec:5.4.2.8	polygalacturonate 4-alpha-galacturonosyltransferase 10.1016/0003-9861(66)90051-8; phosphomannomutase 10.1016/j.cj.2014.07.003	ko:K13648	alpha-1,4-galacturonosyltransferase (GAUT) 10.1007/s00709-020-01508-x
6	Biotin metabolism	ec:1.1.1.100	3-oxoacyl-[acyl-carrier-protein] reductase 10.1016/j.scienta.2020.109388	ko:K00059	3-oxoacyl-[acyl-carrier protein] reductase (OAR1) 10.1016/j.scienta.2020.109388
7	Glycine, serine and threonine metabolism	ec:1.1.1.81	hydroxypyruvate reductase 10.1016/j.envexpbot.2018.10.024		
8	Glutathione metabolism	ec:1.11.1.9	glutathione peroxidase 10.1371/journal.pone.0073989 10.1080/17429145.2018.1458913		
9	Pentose and glucuronate interconversions			ko:K00008	L-iditol 2-dehydrogenase (SORD) 10.1007/s10725-019-00506-1

10	Fatty acid biosynthesis	ec:1.1.1.100;<br><br>ec:2.3.1.85	3-oxoacyl-[acyl-carrier-protein] reductase 10.1016/j.scienta.2020.109388; fatty-acid synthase system 10.1021/np020289t	ko:K00059	3-oxoacyl-[acyl-carrier protein] reductase (OAR1) 10.1016/j.scienta.2020.109388
11	Proteasome			ko:K02738	20S proteasome subunit beta 1 (PSMB6) 10.1093/genetics/149.2.677
12	Biosynthesis of siderophore group nonribosomal peptides	ec:1.3.1.28	2,3-dihydro-2,3-dihydroxybenzoate https://doi.org/10.1021/np020289t		
13	RNA transport			ko:K03238	translation initiation factor 2 subunit 2 (EIF2S2) 10.1071/FP12116 10.1002/pmic.200300570
14	Glyoxylate and dicarboxylate metabolism	ec:1.1.1.79;<br>ec:1.1.1.81	glyoxylate reductase (NADP+) 10.1093/jxb/ern122; hydroxypyruvate reductase 10.1016/j.envexpbot.2018.10.024		
15	RNA degradation			ko:K12581	CCR4-NOT transcription complex subunit 7/8 (CNOT7_8) 10.1186/s12864-018-4727-5 10.1038/cr.2008.317
16	Butanoate metabolism	ec:1.1.1.30	3-hydroxybutyrate dehydrogenase 10.1104/pp.47.6.750		
17	Ascorbate and aldarate metabolism	ec:1.1.1.122	D-threo-aldose 1-dehydrogenase 10.1007/s13562-021-00714-9		
18	Photosynthesis			ko:K02635;<br>ko:K02704	cytochrome b6 (petB) 10.1007/s10535-012-0290-0 ; photosystem II CP47 chlorophyll apoprotein (psbB) 10.3389/fpls.2016.00168
19	Fructose and mannose metabolism	ec:1.1.1.122;<br>ec:5.4.2.8	D-threo-aldose 1-dehydrogenase 10.1007/s13562-021-00714-9; phosphomannomutase	ko:K00008	L-iditol 2-dehydrogenase (SORD) 10.1007/s10725-019-00506-1

			10.1016/j.cj.2014.07.003		
20	Arachidonic acid metabolism	ec:1.11.1.9	glutathione peroxidase 10.1371/journal.pone.0073989		
cluster 4	Pathway	Enzymes Codes	Enzymes Name	KEGG Orthologs	KEGG Orthologs Name
1	RNA polymerase			ko:K03014	DNA-directed RNA polymerases I, II, and III subunit RPABC2 10.5958/2348-7542.2014.00139.9
2	Biosynthesis of unsaturated fatty acids			ko:K01068	acyl-coenzyme A thioesterase 1/2/4 (ACOT1_2_4) 10.3389/fgene.2021.757920
3	Cell cycle			ko:K06636;<br>ko:K06669	structural maintenance of chromosome 1 (SMC1);<br>structural maintenance of chromosome 3 (SMC3) 10.1080/15384101.2021.1966584
4	Biosynthesis of cofactors			ko:K01919	glutamate--cysteine ligase (gshA) 10.1016/j.foodres.2022.111548
5	Terpenoid backbone biosynthesis	ec:5.3.3.2	isopentenyl-diphosphate Delta-isomerase 10.3389/fpls.2021.755788		
6	Starch and sucrose metabolism	ec:3.2.1.2	beta-amylase 10.1016/j.plaphy.2009.05.005	ko:K16055	trehalose 6-phosphate synthase/phosphatase (TPS) 10.1007/s12041-015-0495-z
7	Glycerophospholipid metabolism	ec:2.1.1.71;<br>ec:2.1.1.17	phosphatidyl-N-methylethanolamine N-methyltransferase;<br>phosphatidylethanolamine N-methyltransferase 10.1093/jxb/erac049		
8	Metabolism of xenobiotics by cytochrome P450	ec:2.5.1.18	glutathione S-transferase 10.3389/fpls.2018.01836		
9	Glutathione metabolism	ec:2.5.1.18;<br>ec:6.3.2.2	glutathione S-transferase 10.3389/fpls.2018.01836;<br>glutamylcysteine synthetase	ko:K01919	glutamate--cysteine ligase (gshA) 10.1016/j.foodres.2022.111548
10	Fatty acid elongation			ko:K01068	acyl-coenzyme A thioesterase 1/2/4 (ACOT1_2_4) 10.3389/fgene.2021.757920

11	Protein processing in endoplasmic reticulum			ko:K08057	calreticulin (CALR)10.3389/fpls.2017.01086
12	Ribosome			ko:K02950;<br><br>ko:K02946	small subunit ribosomal protein S12 (RP-S12)<br>10.2174/0929866523666161025<br>small subunit ribosomal protein S10 (RP-S10)10.3390/ijms160920913
13	Peroxisome			ko:K11517	(S)-2-hydroxy-acid oxidase (HAO)<br>10.1007/s11103-019-00866-2<br>10.3390/plants11050700
14	Phagosome			ko:K02150;<br><br>ko:K08057	V-type H+-transporting ATPase subunit E (ATPeV1E)<br>10.1023/A:1022341612852<br>calreticulin (CALR)10.3389/fpls.2017.01086
15	Cytosolic DNA-sensing pathway			ko:K03014	DNA-directed RNA polymerases I, II, and III subunit RPABC2<br>10.5958/2348-7542.2014.00139.9
16	Glyoxylate and dicarboxylate metabolism	ec:1.1.3.15	(S)-2-hydroxy-acid oxidase<br>10.1007/s11103-019-00866-2<br>10.3390/plants11050700	ko:K11517	(S)-2-hydroxy-acid oxidase (HAO)<br>10.1007/s11103-019-00866-2<br>10.3390/plants11050700
17	Aminoacyl-tRNA biosynthesis	ec:6.1.1.20	phenylalanyl-tRNA synthetase		
18	Plant-pathogen interaction			ko:K02358	elongation factor Tu (tuf)<br>10.1016/j.jplph.2007.03.003
19	Tyrosine metabolism	ec:5.2.1.2	maleylacetoacetate isomerase<br>10.1016/j.plantsci.2006.04.008		
20	Ubiquinone and other terpenoid-quinone biosynthesis	ec:1.6.5.2	NAD(P)H dehydrogenase (quinone)<br>10.1093/mp/sst115<br>10.3389/fpls.2016.00383		
21	Photosynthesis	ec:7.1.1.6	plastoquinol/plastocyanin oxidoreductase<br>10.1021/bi4013534	ko:K08901;<br><br>ko:K02703;	photosystem II oxygen-evolving enhancer protein 3 (psbQ)<br>10.1016/j.envexpbot.2016.12.001

				ko:K02636	10.3390/plants7040100; photosystem II P680 reaction center D1 protein (psbA) 10.3390/plants7040100; cytochrome b6-f complex iron-sulfur subunit (petC) 10.1016/j.jprot.2013.03.009
22	Cutin, suberine and wax biosynthesis	ec:4.1.99.5	aldehyde oxygenase 10.1194/jlr.D080283		
23	Cysteine and methionine metabolism	ec:6.3.2.2	gamma-glutamylcysteine synthetase 10.1007/s11738-011-0746-4	ko:K01919	glutamate--cysteine ligase (gshA) 10.1016/j.foodres.2022.111548
24	Oxidative phosphorylation	ec:7.1.1.9; ec:7.1.2.1; ec:7.1.1.2	cytochrome-c oxidase 10.1016/j.plantsci.2005.08.014 ; proton-translocating ATPase; ubiquinone reductase	ko:K02150	V-type H+-transporting ATPase subunit E (ATPeV1E) 10.1023/A:1022341612852
cluster 6	Pathway	Enzymes Codes	Enzymes Name	KEGG Orthologs	KEGG Orthologs Name
1	Phosphatidylinositol signaling system			ko:K19801	phosphatidylinositol 4-kinase B (PI4KB) 10.1093/jxb/ert133 10.1016/j.fgb.2020.103443
2	Spliceosome			ko:K12822	RNA-binding protein 25 (RBM25) 10.1016/j.tplants.2017.09.019
3	Lysosome			ko:K02154	V-type H+-transporting ATPase subunit a (ATPeV0A) 10.1007/s00425-016-2488-4
4	Protein processing in endoplasmic reticulum			ko:K04079	molecular chaperone HtpG (HSP90A) 10.1007/s13562-012-0106-5
5	Basal transcription factors			ko:K03125	transcription initiation factor TFIID subunit 1 (TAF1) 10.1111/tpj.13020
6	Ribosome			ko:K02904	large subunit ribosomal protein L29 (RP-L29) 10.1016/j.jprot.2020.104097

7	Phagosome			ko:K02154	V-type H+-transporting ATPase subunit a (ATPeV0A) 10.1007/s00425-016-2488-4
8	NOD-like receptor signaling pathway			ko:K04079	molecular chaperone HtpG (HSP90A) 10.1007/s13562-012-0106-5
9	RNA degradation			ko:K12581	CCR4-NOT transcription complex subunit 7/8 (CNOT7_8) 10.1186/s12864-018-4727-5 10.1038/cr.2008.317
10	Inositol phosphate metabolism			ko:K19801	phosphatidylinositol 4-kinase B (PI4KB) 10.1093/jxb/ert133 10.1016/j.fgb.2020.103443
11	Plant-pathogen interaction			ko:K04079	molecular chaperone HtpG (HSP90A) 10.1007/s13562-012-0106-5
12	Oxidative phosphorylation			ko:K02132; ko:K02154	F-type H+-transporting ATPase subunit alpha (ATPeF1A) 10.1186/s12870-020-02552-8 V-type H+-transporting ATPase subunit a (ATPeV0A) 10.1007/s00425-016-2488-4
cluster 7	Pathway	Enzymes Codes	Enzymes Name	KEGG Orthologs	KEGG Orthologs Name
1	alpha-Linolenic acid metabolism			ko:K00454	lipoxygenase (LOX2S) 10.3390/ijms21020673
2	Linoleic acid metabolism			ko:K00454	lipoxygenase (LOX2S) 10.3390/ijms21020673
3	Oxidative phosphorylation			ko:K03955	NADH dehydrogenase (ubiquinone) 1 alpha/beta subcomplex 1 (NDUFAB1) 10.3390/ijms22157905
4	Phenylpropanoid biosynthesis			ko:K00430	peroxidase 10.1016/S1673-8527(08)60101-0
cluster 8	Pathway	Enzymes Codes	Enzymes Name	KEGG Orthologs	KEGG Orthologs Name

1	Spliceosome			ko:K03283	heat shock 70kDa protein 1/2/6/8 (HSPA1s) 10.1038/s41598-021-89140-4
2	MAPK signaling pathway			ko:K03283	heat shock 70kDa protein 1/2/6/8 (HSPA1s) 10.1038/s41598-021-89140-4
3	Protein processing in endoplasmic reticulum			ko:K03283	heat shock 70kDa protein 1/2/6/8 (HSPA1s) 10.1038/s41598-021-89140-4
4	Endocytosis			ko:K12191;<br><br>ko:K03283	charged multivesicular body protein 2A (CHMP2A) 10.1016/j.plantsci.2019.01.017<br>heat shock 70kDa protein 1/2/6/8 (HSPA1s) 10.1038/s41598-021-89140-4
5	Metabolism of xenobiotics by cytochrome P450	ec:2.5.1.18	glutathione S-transferase 10.3389/fpls.2018.01836		
6	Estrogen signaling pathway			ko:K03283	heat shock 70kDa protein 1/2/6/8 (HSPA1s) 10.1038/s41598-021-89140-4
7	Glutathione metabolism	ec:2.5.1.18	glutathione S-transferase 10.3389/fpls.2018.01836		
cluster 9	Pathway	Enzymes Codes	Enzymes Name	KEGG Orthologs	KEGG Orthologs Name
1	Ribosome			ko:K02918	large subunit ribosomal protein L35e (RPL35) 10.1093/pcp/pcw074
2	RNA transport			ko:K03254	translation initiation factor 3 subunit A (EIF3A)https://doi.org/10.1016/j.jprot.2011.02 .006

Table S2. The result of time-course expression and KEGG analysis in nine clusters was presented with contig ID (based on TSA: GJUY00000000.1) and numbers of contigs (based on CoutTable, AnnotationTable DOI: 10.17632/p66v4yxbtp.1 and Time-courseTable DOI: 10.17632/xvvscxpz6w.1).

	cluster1								
	Pathway	Pathway ID	#EC	#KO	Sequences	0min	15min	24h	48h
1	Diterpenoid biosynthesis	ko00904	1	2	TRINITY_DN10180_c0_g1	53	178	258	749
2	Plant hormone signal transduction	ko04075	0	1	TRINITY_DN5387_c0_g1	0	37	561	1235
3	Monoterpenoid biosynthesis	ko00902	0	1	TRINITY_DN424_c0_g1	199	218	313	487
4	Stilbenoid, diarylheptanoid and gingerol biosynthesis	ko00945	0	2	TRINITY_DN7321_c0_g1; TRINITY_DN601_c0_g1	161	230	1099	4651
5	Glycosphingolipid biosynthesis - globo and isoglobo series	ko00603	1	0	TRINITY_DN2679_c0_g1	108	113	117	179
6	MAPK signaling pathway - plant	ko04016	0	1	TRINITY_DN5387_c0_g1	0	37	561	1235
7	Polyketide sugar unit biosynthesis	ko00523	1	0	TRINITY_DN414_c0_g1	420	838	727	1636
8	Flavonoid biosynthesis	ko00941	0	1	TRINITY_DN7321_c0_g1	130	127	192	299
9	Phenylpropanoid biosynthesis	ko00940	1	3	TRINITY_DN7321_c0_g1; TRINITY_DN7152_c0_g1; TRINITY_DN991_c0_g1	2150	2707	3559	9337
10	Pyruvate metabolism	ko00620	2	0	TRINITY_DN502_c0_g1	407	502	498	652
11	Ether lipid metabolism	ko00565	1	0	TRINITY_DN6833_c0_g1	70	105	86	277
12	Amino sugar and nucleotide sugar metabolism	ko00520	1	0	TRINITY_DN202_c0_g1	3879	8193	12342	23057
13	Porphyrin and chlorophyll metabolism	ko00860	1	0	TRINITY_DN6833_c0_g1	70	105	86	277
14	Metabolism of xenobiotics by cytochrome P450	ko00980	1	1	TRINITY_DN9964_c0_g1; TRINITY_DN185_c0_g1	2738	4036	5171	10097
15	Glycerolipid metabolism	ko00561	1	0	TRINITY_DN8397_c0_g1	108	113	117	179
16	Glutathione metabolism	ko00480	2	1	TRINITY_DN9964_c0_g1; TRINITY_DN185_c0_g1; TRINITY_DN991_c0_g1	4702	6332	7687	16626

17	Pentose and glucuronate interconversions	ko00040	1	0	TRINITY_DN8397_c0_g1	0	17	81	385
18	Tryptophan metabolism	ko00380	0	1	TRINITY_DN7152_c0_g1	56	284	851	2506
19	Nitrogen metabolism	ko00910	1	0	TRINITY_DN2675_c0_g1	243	400	487	829
20	Carbon fixation in photosynthetic organisms	ko00710	1	0	TRINITY_DN502_c0_g1	407	502	498	652
21	Plant-pathogen interaction	ko04626	0	2	TRINITY_DN5387_c0_g1; TRINITY_DN7016_c0_g1	64	137	665	1403
22	Ascorbate and aldarate metabolism	ko00053	2	0	TRINITY_DN8397_c0_g1; TRINITY_DN991_c0_g1	1964	2313	2597	6914
23	Galactose metabolism	ko00052	1	0	TRINITY_DN8397_c0_g1	0	17	81	385
24	Fructose and mannose metabolism	ko00051	2	0	TRINITY_DN8397_c0_g1	0	17	81	385
	cluster2								
	Pathway	Pathway ID	#EC	#KO	Sequences	0min	15min	24h	48h
1	Ribosome	ko03010	0	1	TRINITY_DN669_c0_g1	489	1121	1335	615
2	Glycerophospholipid metabolism	ko00564	1	0	TRINITY_DN4082_c0_g1	259	496	606	345
3	Porphyrin and chlorophyll metabolism	ko00860	1	0	TRINITY_DN4725_c0_g1	122	458	622	217
4	Aminoacyl-tRNA biosynthesis	ko00970	0	1	TRINITY_DN669_c0_g1	489	1121	1335	615
5	Metabolism of xenobiotics by cytochrome P450	ko00980	1	0	TRINITY_DN3589_c0_g1	322	434	450	327
6	Glycerolipid metabolism	ko00561	1	0	TRINITY_DN4082_c0_g1	259	496	606	345
7	Purine metabolism	ko00230	1	0	TRINITY_DN844_c0_g1	1196	2035	2189	1253
8	Pyrimidine metabolism	ko00240	1	0	TRINITY_DN844_c0_g1	1196	2035	2189	1253
9	Tyrosine metabolism	ko00350	1	0	TRINITY_DN3589_c0_g1	322	434	450	327
10	Glutathione metabolism	ko00480	1	0	TRINITY_DN3589_c0_g1	322	434	450	327
11	Cysteine and methionine metabolism	ko00270	1	0	TRINITY_DN58_c0_g1	884	2058	2748	1464
	cluster3								
	Pathway	Pathway ID	#EC	#KO	Sequences	0min	15min	24h	48h

1	Biosynthesis of cofactors	ko01240	0	1	TRINITY_DN1641_c0_g1	478	515	481	350
2	Phenylpropanoid biosynthesis	ko00940	1	0	TRINITY_DN801_c0_g1	435	523	406	125
3	Pyruvate metabolism	ko00620	1	0	TRINITY_DN1067_c0_g1	419	336	373	187
4	Glycerophospholipid metabolism	ko00564	1	1	TRINITY_DN6517_c0_g1	322	264	286	112
5	Amino sugar and nucleotide sugar metabolism	ko00520	2	1	TRINITY_DN5508_c0_g1; TRINITY_DN4810_c0_g1	511	454	463	299
6	Biotin metabolism	ko00780	1	1	TRINITY_DN1641_c0_g1	478	515	481	350
7	Glycine, serine and threonine metabolism	ko00260	1	0	TRINITY_DN1067_c0_g1	419	336	373	187
8	Glutathione metabolism	ko00480	1	0	TRINITY_DN3363_c0_g1	955	783	749	427
9	Pentose and glucuronate interconversions	ko00040	0	1	TRINITY_DN801_c0_g1	435	523	406	125
10	Fatty acid biosynthesis	ko00061	2	1	TRINITY_DN1641_c0_g1	478	515	481	350
11	Proteasome	ko03050	0	1	TRINITY_DN2484_c0_g1	702	649	686	438
12	Biosynthesis of siderophore group nonribosomal peptides	ko01053	1	0	TRINITY_DN1641_c0_g1	478	515	481	350
13	RNA transport	ko03013	0	1	TRINITY_DN973_c0_g1	885	933	885	666
14	Glyoxylate and dicarboxylate metabolism	ko00630	2	0	TRINITY_DN1067_c0_g1	419	336	373	187
15	RNA degradation	ko03018	0	1	TRINITY_DN6783_c0_g1	1221	1180	1141	564
16	Butanoate metabolism	ko00650	1	0	TRINITY_DN2069_c0_g1	1127	922	988	663
17	Ascorbate and aldarate metabolism	ko00053	1	0	TRINITY_DN391_c0_g1; TRINITY_DN7705_c0_g1	3097	2538	2880	1395
18	Photosynthesis	ko00195	0	2	TRINITY_DN9926_c0_g1	63922	51318	60502	20877
19	Fructose and mannose metabolism	ko00051	2	1	TRINITY_DN801_c0_g1; TRINITY_DN391_c0_g1; TRINITY_DN4810_c0_g1; TRINITY_DN7705_c0_g1	3728	3222	3454	1593
20	Arachidonic acid metabolism	ko00590	1	0	TRINITY_DN3363_c0_g1	955	783	749	427
	cluster4								
	Pathway	Pathway ID	#EC	#KO	Sequences	0min	15min	24h	48h

1	RNA polymerase	ko03020	0	1	TRINITY_DN466_c0_g1	3058	2432	2383	2061
2	Biosynthesis of unsaturated fatty acids	ko01040	0	1	TRINITY_DN7573_c0_g1	392	289	244	122
3	Cell cycle	ko04110	0	2	TRINITY_DN8524_c0_g1; TRINITY_DN2736_c0_g1	683	555	421	301
4	Biosynthesis of cofactors	ko01240	0	1	TRINITY_DN1559_c0_g1	1274	806	879	530
5	Terpenoid backbone biosynthesis	ko00900	1	0	TRINITY_DN10100_c0_g1	195	76	82	25
6	Starch and sucrose metabolism	ko00500	1	1	TRINITY_DN80_c0_g1; TRINITY_DN4973_c0_g1	2597	2039	1301	971
7	Glycerophospholipid metabolism	ko00564	2	0	TRINITY_DN7833_c0_g1	216	171	135	83
8	Metabolism of xenobiotics by cytochrome P450	ko00980	1	0	TRINITY_DN5210_c0_g1	237	171	124	106
9	Glutathione metabolism	ko00480	2	1	TRINITY_DN5210_c0_g1; TRINITY_DN1559_c0_g1	1511	977	1003	636
10	Fatty acid elongation	ko00062	0	1	TRINITY_DN7573_c0_g1	392	289	244	122
11	Protein processing in endoplasmic reticulum	ko04141	0	1	TRINITY_DN2831_c0_g1	454	258	272	173
12	Ribosome	ko03010	0	2	TRINITY_DN10138_c0_g1; TRINITY_DN27_c0_g1	421836	141286	119227	21170
13	Peroxisome	ko04146	0	1	TRINITY_DN41_c0_g1	9788	5775	4465	2387
14	Phagosome	ko04145	0	2	TRINITY_DN2831_c0_g1; TRINITY_DN2386_c0_g1	1018	549	557	339
15	Cytosolic DNA-sensing pathway	ko04623	0	1	TRINITY_DN466_c0_g1	3058	2432	2383	2061
16	Glyoxylate and dicarboxylate metabolism	ko00630	1	1	TRINITY_DN41_c0_g1	9788	5775	4465	2387
17	Aminoacyl-tRNA biosynthesis	ko00970	1	0	TRINITY_DN2707_c0_g1	477	402	302	215
18	Plant-pathogen interaction	ko04626	0	1	TRINITY_DN1922_c0_g1	7341	4590	3197	2291
19	Tyrosine metabolism	ko00350	1	0	TRINITY_DN5210_c0_g1	237	171	124	106
20	Ubiquinone and other terpenoid-quinone biosynthesis	ko00130	1	0	TRINITY_DN10175_c0_g1	1861	985	702	383
21	Photosynthesis	ko00195	1	3	TRINITY_DN80_c0_g1; TRINITY_DN10252_c0_g1;	50639	18943	21523	7960

					TRINITY_DN99_c0_g1				
22	Cutin, suberine and wax biosynthesis	ko00073	1	0	TRINITY_DN6773_c0_g1	171	67	80	29
23	Cysteine and methionine metabolism	ko00270	1	1	TRINITY_DN1559_c0_g1	1274	806	879	530
24	Oxidative phosphorylation	ko00190	3	1	TRINITY_DN2386_c0_g1; TRINITY_DN10172_c0_g1; TRINITY_DN10175_c0_g1	3559	2135	1640	1053
	cluster6								
	Pathway	Pathway ID	#EC	#KO	Sequences	0min	15min	24h	48h
1	Phosphatidylinositol signaling system	ko04070	0	1	TRINITY_DN3650_c0_g1	666	570	416	230
2	Spliceosome	ko03040	0	1	TRINITY_DN6566_c0_g1	442	489	405	186
3	Lysosome	ko04142	0	1	TRINITY_DN3101_c0_g1	997	1039	871	704
4	Protein processing in endoplasmic reticulum	ko04141	0	1	TRINITY_DN767_c0_g1; TRINITY_DN1230_c0_g1	4319	4357	3641	2002
5	Basal transcription factors	ko03022	0	1	TRINITY_DN6772_c0_g1	191	201	154	76
6	Ribosome	ko03010	0	1	TRINITY_DN4512_c0_g1	394	347	255	138
7	Phagosome	ko04145	0	1	TRINITY_DN3101_c0_g1	997	1039	871	704
8	NOD-like receptor signaling pathway	ko04621	0	1	TRINITY_DN767_c0_g1; TRINITY_DN1230_c0_g1	4319	4357	3641	2002
9	RNA degradation	ko03018	0	1	TRINITY_DN7977_c0_g1	229	220	184	114
10	Inositol phosphate metabolism	ko00562	0	1	TRINITY_DN3650_c0_g1	666	570	416	230
11	Plant-pathogen interaction	ko04626	0	1	TRINITY_DN767_c0_g1; TRINITY_DN1230_c0_g1	4319	4357	3641	2002
12	Oxidative phosphorylation	ko00190	0	2	TRINITY_DN2836_c0_g1; TRINITY_DN3101_c0_g1	2459	2420	1957	1458
	cluster7								
	Pathway	Pathway ID	#EC	#KO	Sequences	0min	15min	24h	48h
1	alpha-Linolenic acid metabolism	ko00592	0	1	TRINITY_DN62_c0_g1	9572	2480	238	98
2	Linoleic acid metabolism	ko00591	0	1	TRINITY_DN62_c0_g1	9572	2480	238	98

3	Oxidative phosphorylation	ko00190	0	1	TRINITY_DN1772_c0_g1	519	290	231	202
4	Phenylpropanoid biosynthesis	ko00940	0	1	TRINITY_DN898_c0_g1	928	428	343	580
	cluster8								
	Pathway	Pathway ID	#EC	#KO	Sequences	0min	15min	24h	48h
1	Spliceosome	ko03040	0	1	TRINITY_DN296_c0_g1	548	2341	3793	2626
2	MAPK signaling pathway	ko04010	0	1	TRINITY_DN296_c0_g1	548	2341	3793	2626
3	Protein processing in endoplasmic reticulum	ko04141	0	1	TRINITY_DN296_c0_g1	548	2341	3793	2626
4	Endocytosis	ko04144	0	2	TRINITY_DN296_c0_g1; TRINITY_DN1213_c0_g1	872	2817	4320	3147
5	Metabolism of xenobiotics by cytochrome P450	ko00980	1	0	TRINITY_DN487_c0_g1; TRINITY_DN8548_c0_g1	766	1277	1446	1164
6	Glutathione metabolism	ko00480	1	0	TRINITY_DN487_c0_g1; TRINITY_DN8548_c0_g1	766	1277	1446	1164
	cluster9								
	Pathway	Pathway ID	#EC	#KO	Sequences	0min	15min	24h	48h
1	Ribosome	ko03010	0	1	TRINITY_DN714_c0_g1	1081	1509	1621	908
2	RNA transport	ko03013	0	1	TRINITY_DN7096_c0_g1	213	291	309	172

Table S3. Primers of real-time PCR for the investigated gene expression were designed based on contigs of transcript dataset (TSA: GJUY00000000.1) using Primer3Plus software. Housekeeper genes were designed based on sequences of *T. aestivum* in NCBI dataset. Investigated contigs were blasted to NCBI database to control identification of sequences.

Name	Name	Number of contig in TSA	Accession number of NCBI	Sequence (5'-3')	Amplicon size (bp)
TUBB1	β-tubulin1	-	U76744.1	F- agtggatcccgaacaacgtc<br>R- acacgcctgaacatctcctg	115
UB1	ubiquitin	-	AY297059.2	F-ggagtccacccttcacttgg<br>R- agacacaggcaccattcgag	130
PR1	pathogenesis-related1	TRINITY_DN10251_c0_g1	AJ007348.1	F- agtccctcgttccttgcatg<br>R- ttatttactcgcccagcccc	90
PR1-3	pathogenesis-related1-3	TRINITY_DN4265_c0_g1	XM_044587903.1	F- atcacctgcaactacgagcc<br>R- atgtactgcgaagacgtgca	80
PR4	pathogenesis-related4 (wheatwin-2)	TRINITY_DN82_c0_g1	XM_044503879.1	F- caagatcgacaccaacggga<br>R- cgtaatctagtcgcggcagt	85
PR5	pathogenesis-related5 (thaumatin)	TRINITY_DN967_c0_g1	XM_044569611.1	F- ccttgccatggacttctcgt<br>R- gttggggtgttggtaggctt	91
NCED3	9-cis-epoxycarotenoid dioxygenase	TRINITY_DN2829_c0_g1	XM_037580089.1	F- gcttcagtccacaccagtgt<br>R- tctgtagaagcaagcaccgg	146
HVA22	HVA22-like protein	TRINITY_DN541_c0_g1	XM_044539374.1	F- tggcttgtggtgttttcttgg<br>R- agccacccaccatctagcta	140
PUB19	U-box domain-containing protein19	TRINITY_DN897_c0_g1	XM_044537131.1	F- agcgtgatggatacgtgacc<br>R- cacaaggaatggtggggagg	101
LEA31	late embryogenesis abundant protein 31	TRINITY_DN6620_c0_g1	XM_044583651.1	F- gagcatcctgtcgagtgagg<br>R- cgggccagcactgttatttg	85
HOX24	homeobox-leucine zipper protein	TRINITY_DN7677_c0_g1	XM_044560688.1	F- tagggatcccacatgcaagc<br>R- cagagtcgacggagcagatc	85
DREB1G	dehydration-responsive element-binding protein 1G	TRINITY_DN1950_c0_g1	XM_044560734.1	F- ttggtagatggtgcacgctt<br>R- aacaccgacgaagacactcc	125
MYBS1	transcription factor	TRINITY_DN2432_c0_g1	XM_044499707.1	F- tttgctagtggcgtgctaca<br>R- ggatggatcggtgatggcat	94
ZAT12	zinc finger protein	TRINITY_DN5332_c0_g1	XM_037575759.1	F- cgtctaggtagctggatctgc<br>R- gcaaaacgtactctatcgaaccg	90

Table S4. Real-time PCR datasets were presented for the investigated genes. Data were analysed by the ΔΔCt method.

Well	Fluor	Target	Content	Sample	Cq	Cq Mean	Cq Std. Dev
A02	SYBR	TUB	Unkn-05	1_Ctrl	26,91	26,97	0,081
B02	SYBR	TUB	Unkn-05	1_Ctrl	27,02	26,97	0,081
C02	SYBR	TUB	Unkn-06	2_15m	26,59	26,53	0,091
D02	SYBR	TUB	Unkn-06	2_15m	26,47	26,53	0,091
E02	SYBR	TUB	Unkn-07	3_24h	26,27	26,19	0,114
F02	SYBR	TUB	Unkn-07	3_24h	26,11	26,19	0,114
G02	SYBR	TUB	Unkn-08	4_48h	26,56	26,50	0,090
H02	SYBR	TUB	Unkn-08	4_48h	26,44	26,50	0,090
A03	SYBR	UB	Unkn-09	1_Ctrl	25,66	25,54	0,174
B03	SYBR	UB	Unkn-09	1_Ctrl	25,41	25,54	0,174
C03	SYBR	UB	Unkn-10	2_15m	24,74	24,72	0,030
D03	SYBR	UB	Unkn-10	2_15m	24,70	24,72	0,030
E03	SYBR	UB	Unkn-11	3_24h	24,44	24,45	0,026
F03	SYBR	UB	Unkn-11	3_24h	24,47	24,45	0,026
G03	SYBR	UB	Unkn-12	4_48h	25,02	24,91	0,156
H03	SYBR	UB	Unkn-12	4_48h	24,80	24,91	0,156
A05	SYBR	DREB	Unkn-17	1_Ctrl	28,50	28,63	0,181
B05	SYBR	DREB	Unkn-17	1_Ctrl	28,76	28,63	0,181
C05	SYBR	DREB	Unkn-18	2_15m	29,79	29,75	0,056
D05	SYBR	DREB	Unkn-18	2_15m	29,71	29,75	0,056
E05	SYBR	DREB	Unkn-19	3_24h	32,56	32,48	0,117
F05	SYBR	DREB	Unkn-19	3_24h	32,40	32,48	0,117
G05	SYBR	DREB	Unkn-20	4_48h	23,68	23,70	0,030
H05	SYBR	DREB	Unkn-20	4_48h	23,73	23,70	0,030
A07	SYBR	HOX24	Unkn-25	1_Ctrl	28,15	27,97	0,262
B07	SYBR	HOX24	Unkn-25	1_Ctrl	27,78	27,97	0,262

C07	SYBR	HOX24	Unkn-26	2_15m	24,04	24,05	0,016
D07	SYBR	HOX24	Unkn-26	2_15m	24,06	24,05	0,016
E07	SYBR	HOX24	Unkn-27	3_24h	25,05	25,03	0,026
F07	SYBR	HOX24	Unkn-27	3_24h	25,01	25,03	0,026
G07	SYBR	HOX24	Unkn-28	4_48h	29,02	28,92	0,148
H07	SYBR	HOX24	Unkn-28	4_48h	28,81	28,92	0,148
A09	SYBR	LEA31	Unkn-33	1_Ctrl	29,16	29,23	0,099
B09	SYBR	LEA31	Unkn-33	1_Ctrl	29,30	29,23	0,099
C09	SYBR	LEA31	Unkn-34	2_15m	25,63	25,61	0,023
D09	SYBR	LEA31	Unkn-34	2_15m	25,60	25,61	0,023
E09	SYBR	LEA31	Unkn-35	3_24h	26,81	26,83	0,030
F09	SYBR	LEA31	Unkn-35	3_24h	26,85	26,83	0,030
G09	SYBR	LEA31	Unkn-36	4_48h	27,11	27,13	0,030
H09	SYBR	LEA31	Unkn-36	4_48h	27,15	27,13	0,030
A10	SYBR	MYB	Unkn-37	1_Ctrl	27,73	27,97	0,349
B10	SYBR	MYB	Unkn-37	1_Ctrl	28,22	27,97	0,349
C10	SYBR	MYB	Unkn-38	2_15m	24,23	24,26	0,048
D10	SYBR	MYB	Unkn-38	2_15m	24,30	24,26	0,048
E10	SYBR	MYB	Unkn-39	3_24h	24,54	24,56	0,031
F10	SYBR	MYB	Unkn-39	3_24h	24,58	24,56	0,031
G10	SYBR	MYB	Unkn-40	4_48h	27,97	27,89	0,110
H10	SYBR	MYB	Unkn-40	4_48h	27,81	27,89	0,110
A11	SYBR	NCED3	Unkn-41	1_Ctrl	29,06	28,88	0,248
B11	SYBR	NCED3	Unkn-41	1_Ctrl	28,71	28,88	0,248
C11	SYBR	NCED3	Unkn-42	2_15m	23,65	23,70	0,064
D11	SYBR	NCED3	Unkn-42	2_15m	23,74	23,70	0,064
F11	SYBR	NCED3	Unkn-43	3_24h	25,41	25,41	0,000
G11	SYBR	NCED3	Unkn-44	4_48h	32,29	32,63	0,473
H11	SYBR	NCED3	Unkn-44	4_48h	32,96	32,63	0,473
A14	SYBR	PR4	Unkn-53	1_Ctrl	25,03	25,23	0,289

B14	SYBR	PR4	Unkn-53	1_Ctrl	25,44	25,23	0,289
C14	SYBR	PR4	Unkn-54	2_15m	24,29	24,09	0,288
D14	SYBR	PR4	Unkn-54	2_15m	23,88	24,09	0,288
E14	SYBR	PR4	Unkn-55	3_24h	20,90	20,93	0,048
F14	SYBR	PR4	Unkn-55	3_24h	20,97	20,93	0,048
G14	SYBR	PR4	Unkn-56	4_48h	21,89	21,88	0,022
H14	SYBR	PR4	Unkn-56	4_48h	21,86	21,88	0,022
A15	SYBR	PR5	Unkn-57	1_Ctrl	24,57	24,66	0,116
B15	SYBR	PR5	Unkn-57	1_Ctrl	24,74	24,66	0,116
C15	SYBR	PR5	Unkn-58	2_15m	22,98	23,04	0,095
D15	SYBR	PR5	Unkn-58	2_15m	23,11	23,04	0,095
E15	SYBR	PR5	Unkn-59	3_24h	21,25	21,26	0,011
F15	SYBR	PR5	Unkn-59	3_24h	21,27	21,26	0,011
G15	SYBR	PR5	Unkn-60	4_48h	20,33	20,15	0,246
H15	SYBR	PR5	Unkn-60	4_48h	19,98	20,15	0,246
A17	SYBR	PUB19	Unkn-65	1_Ctrl	28,50	28,37	0,186
B17	SYBR	PUB19	Unkn-65	1_Ctrl	28,24	28,37	0,186
C17	SYBR	PUB19	Unkn-66	2_15m	24,72	24,68	0,057
D17	SYBR	PUB19	Unkn-66	2_15m	24,64	24,68	0,057
E17	SYBR	PUB19	Unkn-67	3_24h	25,60	25,58	0,027
F17	SYBR	PUB19	Unkn-67	3_24h	25,57	25,58	0,027
G17	SYBR	PUB19	Unkn-68	4_48h	25,84	25,92	0,110
H17	SYBR	PUB19	Unkn-68	4_48h	26,00	25,92	0,110
A19	SYBR	ZAT12	Unkn-73	1_Ctrl	29,12	29,10	0,021
B19	SYBR	ZAT12	Unkn-73	1_Ctrl	29,09	29,10	0,021
C19	SYBR	ZAT12	Unkn-74	2_15m	25,35	25,29	0,075
D19	SYBR	ZAT12	Unkn-74	2_15m	25,24	25,29	0,075
E19	SYBR	ZAT12	Unkn-75	3_24h	26,41	26,40	0,019
F19	SYBR	ZAT12	Unkn-75	3_24h	26,39	26,40	0,019
G19	SYBR	ZAT12	Unkn-76	4_48h	26,55	26,40	0,206

H19	SYBR	ZAT12	Unkn-76	4_48h	26,26	26,40	0,206
I02	SYBR	TUB	NTC-78	NFW	N/A	0,00	0,000
J02	SYBR	TUB	NTC-78	NFW	N/A	0,00	0,000
I03	SYBR	UB	NTC-79	NFW	N/A	0,00	0,000
J03	SYBR	UB	NTC-79	NFW	N/A	0,00	0,000
I05	SYBR	DREB	NTC-81	NFW	N/A	0,00	0,000
J05	SYBR	DREB	NTC-81	NFW	N/A	0,00	0,000
I07	SYBR	HOX24	NTC-83	NFW	N/A	0,00	0,000
J07	SYBR	HOX24	NTC-83	NFW	38,17	38,17	0,000
I09	SYBR	LEA31	NTC-85	NFW	N/A	0,00	0,000
J09	SYBR	LEA31	NTC-85	NFW	37,65	37,65	0,000
I10	SYBR	MYB	NTC-86	NFW	N/A	0,00	0,000
J10	SYBR	MYB	NTC-86	NFW	N/A	0,00	0,000
I11	SYBR	NCED3	NTC-87	NFW	29,60	29,17	0,611
J11	SYBR	NCED3	NTC-87	NFW	28,74	29,17	0,611
I14	SYBR	PR4	NTC-90	NFW	33,69	36,57	4,075
J14	SYBR	PR4	NTC-90	NFW	39,46	36,57	4,075
I15	SYBR	PR5	NTC-91	NFW	39,61	38,94	0,941
J15	SYBR	PR5	NTC-91	NFW	38,28	38,94	0,941
I17	SYBR	PUB19	NTC-93	NFW	38,94	39,05	0,163
J17	SYBR	PUB19	NTC-93	NFW	39,17	39,05	0,163
I19	SYBR	ZAT12	NTC-95	NFW	N/A	0,00	0,000
J19	SYBR	ZAT12	NTC-95	NFW	37,30	37,30	0,000
A02	SYBR	TUB	Unkn-05	1_Ctrl	26,93	26,98	0,077
B02	SYBR	TUB	Unkn-05	1_Ctrl	27,04	26,98	0,077
C02	SYBR	TUB	Unkn-06	2_15m	26,61	26,55	0,092
D02	SYBR	TUB	Unkn-06	2_15m	26,48	26,55	0,092
E02	SYBR	TUB	Unkn-07	3_24h	26,29	26,21	0,114
F02	SYBR	TUB	Unkn-07	3_24h	26,13	26,21	0,114
G02	SYBR	TUB	Unkn-08	4_48h	26,58	26,52	0,090

H02	SYBR	TUB	Unkn-08	4_48h	26,45	26,52	0,090
A03	SYBR	UB	Unkn-09	1_Ctrl	25,67	25,55	0,175
B03	SYBR	UB	Unkn-09	1_Ctrl	25,43	25,55	0,175
C03	SYBR	UB	Unkn-10	2_15m	24,76	24,74	0,031
D03	SYBR	UB	Unkn-10	2_15m	24,72	24,74	0,031
E03	SYBR	UB	Unkn-11	3_24h	24,45	24,47	0,027
F03	SYBR	UB	Unkn-11	3_24h	24,49	24,47	0,027
G03	SYBR	UB	Unkn-12	4_48h	25,03	24,92	0,152
H03	SYBR	UB	Unkn-12	4_48h	24,81	24,92	0,152
A08	SYBR	HVA22	Unkn-29	1_Ctrl	26,90	26,77	0,188
B08	SYBR	HVA22	Unkn-29	1_Ctrl	26,64	26,77	0,188
C08	SYBR	HVA22	Unkn-30	2_15m	22,17	22,17	0,010
D08	SYBR	HVA22	Unkn-30	2_15m	22,16	22,17	0,010
E08	SYBR	HVA22	Unkn-31	3_24h	24,07	24,08	0,020
F08	SYBR	HVA22	Unkn-31	3_24h	24,09	24,08	0,020
G08	SYBR	HVA22	Unkn-32	4_48h	27,88	27,91	0,037
H08	SYBR	HVA22	Unkn-32	4_48h	27,93	27,91	0,037
A12	SYBR	PR3	Unkn-45	1_Ctrl	32,80	32,55	0,361
B12	SYBR	PR3	Unkn-45	1_Ctrl	32,29	32,55	0,361
C12	SYBR	PR3	Unkn-46	2_15m	31,30	31,29	0,014
D12	SYBR	PR3	Unkn-46	2_15m	31,28	31,29	0,014
E12	SYBR	PR3	Unkn-47	3_24h	28,94	29,03	0,128
F12	SYBR	PR3	Unkn-47	3_24h	29,12	29,03	0,128
G12	SYBR	PR3	Unkn-48	4_48h	28,60	28,58	0,032
H12	SYBR	PR3	Unkn-48	4_48h	28,56	28,58	0,032
A13	SYBR	PR1	Unkn-49	1_Ctrl	26,13	26,05	0,126
B13	SYBR	PR1	Unkn-49	1_Ctrl	25,96	26,05	0,126
C13	SYBR	PR1	Unkn-50	2_15m	23,77	23,81	0,047
D13	SYBR	PR1	Unkn-50	2_15m	23,84	23,81	0,047
E13	SYBR	PR1	Unkn-51	3_24h	21,17	21,12	0,081

F13	SYBR	PR1	Unkn-51	3_24h	21,06	21,12	0,081
G13	SYBR	PR1	Unkn-52	4_48h	19,82	19,70	0,170
H13	SYBR	PR1	Unkn-52	4_48h	19,58	19,70	0,170
A16	SYBR	PR6	Unkn-61	1_Ctrl	36,32	36,47	0,208
B16	SYBR	PR6	Unkn-61	1_Ctrl	36,62	36,47	0,208
C16	SYBR	PR6	Unkn-62	2_15m	36,15	35,38	1,088
D16	SYBR	PR6	Unkn-62	2_15m	34,61	35,38	1,088
E16	SYBR	PR6	Unkn-63	3_24h	34,01	34,04	0,032
F16	SYBR	PR6	Unkn-63	3_24h	34,06	34,04	0,032
G16	SYBR	PR6	Unkn-64	4_48h	34,68	35,26	0,827
H16	SYBR	PR6	Unkn-64	4_48h	35,85	35,26	0,827
I02	SYBR	TUB	NTC-78	NFW	N/A	0,00	0,000
J02	SYBR	TUB	NTC-78	NFW	N/A	0,00	0,000
I03	SYBR	UB	NTC-79	NFW	N/A	0,00	0,000
J03	SYBR	UB	NTC-79	NFW	N/A	0,00	0,000
I08	SYBR	HVA22	NTC-84	NFW	N/A	0,00	0,000
J08	SYBR	HVA22	NTC-84	NFW	38,86	38,86	0,000
I12	SYBR	PR3	NTC-88	NFW	N/A	0,00	0,000
J12	SYBR	PR3	NTC-88	NFW	N/A	0,00	0,000
I13	SYBR	PR1	NTC-89	NFW	35,90	34,02	2,650
J13	SYBR	PR1	NTC-89	NFW	32,15	34,02	2,650
I16	SYBR	PR6	NTC-92	NFW	35,60	36,04	0,614
J16	SYBR	PR6	NTC-92	NFW	36,47	36,04	0,614

Target	Sample	Expression ddCq-method	Expression SEM
PR1	Ctrl	1,00	0,077
	15m	3,06	0,101
	24h	16,00	0,784

	48h	55,52	5,213
PR3	Ctrl	1,00	0,183
	15m	1,55	0,038
	24h	6,00	0,414
	48h	10,71	0,494
PR4	Ctrl	1,00	0,149
	15m	1,43	0,205
	24h	10,35	0,383
	48h	7,00	0,319
PR5	Ctrl	1,00	0,074
	15m	1,98	0,103
	24h	5,53	0,162
	48h	15,52	1,991
PR6	Ctrl	1,00	0,112
	15m	1,38	0,739
	24h	2,84	0,093
	48h	1,58	0,644
NCED3	Ctrl	1,00	0,130
	15m	23,62	0,925
	24h	5,83	0,237
	48h	0,05	0,012
HVA22	Ctrl	1,00	0,103
	15m	15,75	0,384
	24h	3,39	0,103
	48h	0,31	0,015
PUB19	Ctrl	1,00	0,102
	15m	8,39	0,306
	24h	3,63	0,115

	48h	3,74	0,260
LEA31	Ctrl	1,00	0,068
	15m	7,92	0,206
	24h	2,77	0,090
	48h	2,91	0,136
HOX24	Ctrl	1,00	0,137
	15m	9,79	0,243
	24h	4,03	0,127
	48h	0,35	0,030
DREB1G	Ctrl	1,00	0,101
	15m	0,30	0,011
	24h	0,04	0,002
	48h	20,77	0,968
MYB	Ctrl	1,00	0,178
	15m	8,48	0,283
	24h	5,59	0,181
	48h	0,72	0,051
ZAT12	Ctrl	1,00	0,048
	15m	9,08	0,395
	24h	3,42	0,103
	48h	4,45	0,491
